# Sexual harassment in India's medical education: need for accountability

**DOI:** 10.1016/j.lansea.2026.100731

**Published:** 2026-02-17

**Authors:** Srijani Paul, Nilabho Chakraborty, Ashish Pundhir, Venkatesh Karthikeyan, Vithya Sree M

**Affiliations:** a College of Medicine and JNM Hospital, West Bengal University of Health Science, West Bengal, India; b College of Medicine and JNM Hospital, West Bengal University of Health Science, West Bengal, India; c Department of Community Medicine and Family Medicine, All India Institute of Medical Sciences, Kalyani, West Bengal, India; d Department of Community Medicine and Family Medicine, All India Institute of Medical Sciences, Patna, Bihar, India; e Govt Sivagangai Medical College, Sivagangai, Tamil Nadu, India

**Keywords:** Sexual harassment, Workplace violence, Sexual violence, Health personnel, Reporting, India, Medical education, Accountability

## Abstract

Sexual harassment in medical education is a global, structurally embedded problem driven by rigid hierarchical cultures, with trainees disproportionately exposed to higher levels of harassment. Policy recommendations reframe harassment as a public health issue, and call for trauma-informed supports, anti-retaliation protections, and psychologically safe training environments.

Sexual harassment within medical education remains a globally pervasive and underrecognised problem.^1^ Evidence from multiple countries reveals that medical students are exposed to disproportionately high levels of sexual violence compared with trainees in other disciplines, with prevalence estimates ranging from one-third to more than half of students, depending on the setting, gender, and stage of training.^2^, ^3^, ^4^, ^5^ Despite growing global attention to gender-based violence, clinical learning environments remain among the most hierarchically rigid and least reflexive professional spaces, in which behaviour spanning from subtle verbal innuendo to explicit coercive acts are normalised and insufficiently addressed.

Studies from the United States, Belgium, Finland, Brazil, Germany, and Switzerland show consistent patterns: significant proportions of medical trainees, especially women and gender-minority students, report inappropriate comments, coercive advances, unwanted touching, and even severe forms of assault during their training years.^2^^,^^4^^,^^6^, ^7^, ^8^, ^9^, ^10^ In Belgium, nearly 40% of medical students and registrars reported sexual violence, with women being twice as likely to be affected.^6^ Evidence from Brazil shows that more than half of female medical students experienced sexual violence during their course, while in Finland, three-quarters of medical students reported mistreatment, much of it sexual in nature.^9^^,^^10^ Adding to the list, evidence from several lower income- and middle-income countries reveals comparable patterns in academic medicine. In Nigeria, 32.4% of healthcare workers report sexual harassment, while studies from Bangladesh report prevalence of 17.9%, commonly linked to rigid hierarchies, weak reporting mechanisms, and fear of retaliation. In Ghana, prevalence among nurses ranges from 10 to 15%, Chinese studies report 13–20%, and in Sri Lanka, 58.6% of women doctors report experiencing workplace sexual harassment at some point in their careers.^2^^,^^11^, ^12^, ^13^, ^14^ These findings demonstrate that the vulnerability of medical trainees is embedded within systems and requires attention.

India mirrors these patterns, although primary data remains sparse and geographically limited.^3^^,^^15^^,^^16^ A 2025 multicenter online based cross-sectional Indian study, which analysed data from 2021, found that 26% of healthcare workers experienced sexual harassment, In particular-younger women were demonstrated to be disproportionately affected -however the study lacked a clear operational definition for sexual harassment.^3^ Another mixed-method survey across 28 medical colleges in the Indian state of Maharashtra documented a 14.3% incidence of sexual harassment, while 43.2% of respondents reported various sexually offencive behaviours.^15^ Data obtained from offline questionnaire-based cross sectional studies conducted in other states -Karnataka, Punjab, and Uttar Pradesh show broadly consistent findings.^16^, ^17^, ^18^ A study from Banaras Hindu University, located in Varanasi, India reported that none of the participants affected by sexual harassment disclosed their experiences to the police, underscoring potential reporting barriers.^18^ However, the results of Indian studies vary widely without a clear or consistent pattern and should be interpreted cautiously due to their lack of a standardised instrument, in depth analysis and possible sampling and reporting biases^3^^,^^15^, ^16^, ^17^, ^18^ and unclear definition.^3^^,^^17^

This Viewpoint examines how hierarchical vulnerabilities are compounded by weak institutional accountability and inconsistent enforcement within medical training environments in the Indian context, it is noteworthy that existing studies on sexual violence among medical students in India are zonally skewed, with data largely from the in southern and northern areas of India, without disaggregation for caste, socio economic and rural-urban status. Evidence from other higher-education settings demonstrates that intersection of caste and class discrimination places marginalised women, including those belonging to disadvantaged caste groups at heightened risk in India^19^ underscoring the need for regionally inclusive, methodologically robust well-structured research within the medical academia using validated standard questionnaires to comprehensively capture the full scope of the issue.

The rigid hierarchy of medical education concentrates power in senior ranks -senior students, interns, residents, faculty, and consultants who control postings, assessments, and career-defining opportunities.^20^^,^^21^ International research has identified academic reprisal, mentorship loss, and reputational harm as key reporting deterrents. Abusive conduct is rationalised as “rigor” or “tradition,” thereby blurring the boundary between legitimate authority and coercive behaviour.^20^^,^^21^ Across cross-sectional studies, reporting of incidents remains low, with disclosures by affected individuals ranging from 0% to 21%, despite substantial prevalence and increasing exposure as training progresses, in the absence of publicly available or systematically documented Internal Complaints Committee (ICC) data.^18^^,^^20^

Within this wider landscape, the widely publicised rape and murder of an on duty doctor within the hospital premises at R.G. Kar Medical College in Kolkata, India brought into sharp focus failures in workplace safety, institutional accountability, and violence prevention mechanisms in healthcare settings. The incident underscores the persistent safety risks faced by health personnel, barriers to incident reporting, and systemic gaps in protecting healthcare workers, reflecting challenges observed across the country.^22^

Moving from prevalence to lived experience, the hierarchical culture of medical training plays a crucial role. Mental health stigma and fear of “unprofessional” labels deter help-seeking.^20^^,^^21^ Victims suffer from anxiety, depression, hypervigilance, PTSD-like symptoms, exhaustion, creating psychologically unsafe spaces.^7^^,^^23^^,^^24^ Academically, affected individuals avoid postings, skip activities, face concentration lapses, absenteeism, lower grades, and course withdrawals. This erodes research access, mentorship, and career paths, reducing workforce diversity.^20^^,^^21^^,^^23^, ^24^, ^25^

Sexual harassment incurs indirect costs through absenteeism, reduced productivity, workforce attrition, and loss of human capital, cumulatively generating substantial economic drag.^25^

Therefore, recognising sexual harassment as a public health and workforce issue, not merely a disciplinary matter, is essential. Leaders in medical education must model respectful behaviour, confront colleagues who perpetuate harm, and make it clear that academic excellence is inseparable from ethical conduct. Solutions demand strengthening student networks, peer groups, trauma-informed probes, anti-retaliation policies, gender sensitivity training, and psychological safety curricula.^26^^,^^27^ While no publicly available medical-college–specific ICC audit data could be identified, evidence from ICC audits in other higher-education settings indicates low levels of formal reporting and raises concerns about the limited functionality and effectiveness of these mechanisms^28^ and highlights the need for institutions to treat these bodies with greater seriousness; correspondingly, the POSH Act (Prevention of Sexual Harassment at Workplace Act) and the National Medical Commission must provide stringent operational guidelines to support their reform and effective implementation.^29^ By acknowledging the depth of the problem it's important to discuss why we feel the ICC fail structurally. Internal Complaints Committees (ICCs), as mandated under the POSH Act, are structurally compromised by their lack of institutional independence. They are constituted, resourced, and administratively controlled by the very organisations they are expected to hold accountable. In medical colleges, where authority is concentrated in senior faculty and administrators, ICC members are often colleagues, sometimes subordinates of the accused. The assumed procedural equality between complainant and respondent does not exist in hierarchical training environments. Additionally, many ICC members lack training in trauma-informed inquiry leading to hostile questioning and secondary victimisation. Additionally, we believe the creation of a centralised, confidential reporting portal for sexual harassment and assault in medical institutions modelled on the University Grants Commission's Anti-Ragging portal (where “ragging” in the Indian context refers to institutionalised bullying and harassment of junior students by seniors within educational hierarchies),^30^ could address several barriers to people seeking help. A practical precedent exists in India through the Ministry of Women and Child Development's Sexual Harassment electronic Box (SHe-Box), a POSH Act–anchored digital platform that enables centralised complaint submission and tracking while maintaining confidentiality.^31^ Its use of role-restricted access, limited data collection, and controlled information sharing reflects core GDPR (General Data Protection Regulation)-aligned principles of data minimisation, purpose limitation, and confidentiality. Adapting similar safeguards within medical education would permit national-level reporting without compromising privacy or procedural fairness. To be effective, such mechanisms must be coupled with enforceable accountability. Regulatory bodies, including the National Medical Commission, can link compliance to accreditation standards, audits, and administrative or funding-related consequences. Without external oversight, reporting systems risk remaining symbolic. With sustained institutional reforms, use of technology and committing to structural change, medical institutions can begin to rebuild trust and create training environments where every student—regardless of gender—can learn, grow and thrive without fear.

## Contributors

SP: Conceptualisation, investigation, methodology, data curation, project administration, writing—original draft, and writing—review & editing.

NC: Conceptualisation, investigation, methodology, writing—original draft, and writing—review & editing.

AP: Conceptualisation, supervision, and writing—review & editing.

VK: Supervision.

VSM: Writing—review & editing.

All authors have read and approved the final manuscript.

## Declaration of generative AI and AI-assisted technologies in the manuscript preparation process

During the preparation of this work the author(s) used GPT-5 (OpenAI) and Paperpal in order to assist with language editing, grammar correction, and improvement of readability. After using these tools/services, the author(s) reviewed and edited the content as needed and take(s) full responsibility for the content of the published article.

## Declaration of interests

We declare no competing interests.
